# Detection and quantification of *Dioctophyme renale eggs* in dog urine after nephrectomy

**DOI:** 10.1590/S1984-29612024067

**Published:** 2024-11-22

**Authors:** Soliane Carra Perera, Maria Antonieta Machado Pereira da Silva, Gabriela de Almeida Capella, Natália Berne Pinheiro, Maria Elisabeth Aires Berne, Josaine Cristina da Silva Rappeti, Fabrício de Vargas Arigony Braga, Marlete Brum Cleff

**Affiliations:** 1 Grupo de Pesquisa, Ensino e Extensão em Produtos Naturais na Clínica Médica Veterinária, Departamento de Clínicas Veterinária, Faculdade de Veterinária, Universidade Federal de Pelotas – UFPel, Pelotas, RS, Brasil; 2 Projeto Dioctophyme renale em Cães e Gatos, Departamento de Clínicas Veterinária, Faculdade de Veterinária, Universidade Federal de Pelotas – UFPel, Pelotas, RS, Brasil; 3 Departamento de Microbiologia e Parasitologia, Instituto de Biologia, Universidade Federal de Pelotas – UFPel, Pelotas, RS, Brasil; 4 Departamento de Clínicas Veterinária, Faculdade de Veterinária, Universidade Federal de Pelotas – UFPel, Pelotas, RS, Brasil

**Keywords:** Giant kidney worm, dioctophymosis, canids, helminth eggs, environmental contamination, Verme gigante do rim, dioctofimose, canídeos, ovos de helminto, contaminação ambiental

## Abstract

*Dioctophyme renale* is a zoonotic nematode that parasitizes mainly right kidney of domestic and wild canines, and can affect humans, and its eggs are eliminated in urine. The duration of egg dissemination after surgical treatment is unknown. Therefore, the objective of this study was to identify, quantify, and verify the duration of the elimination of *D. renale* eggs in dog urine after the surgical removal of nematodes. The study involved 15 dogs in which female specimens of *D. renale* were detected in kidneys. Urine samples, preferably first-morning urine samples, were collected before and for the first ten days after nephrectomy. For egg quantification, 40 µL samples of urinary sediment were analyzed in triplicate. In laboratory analyses, between 900 and > 6,000 eggs/urine sample were detected in 86.7% of the dogs prior to surgery, and in 40% of the dogs on postoperative day 1. Of the 15 dogs evaluated, 14 (93.3%) eliminated *D. renale* eggs on each of the first ten postoperative days. Egg elimination peaked on postoperative day 1. Our results indicate that dogs can continue to be sources of *D. renale* infection even after the helminths have been removed from a parasitized kidney, underscoring the novelty of these findings and their importance for the One Health approach.

## Resumo

*Dioctophyme renale* é um nematódeo zoonótico que parasita principalmente o rim direito de caninos domésticos e silvestres, podendo parasitar também humanos, sendo seus ovos eliminados pela urina. É desconhecido o período de disseminação de ovos após o tratamento cirúrgico, assim o objetivo desse trabalho foi verificar, quantificar e acompanhar o tempo de eliminação de ovos de *D. renale* na urina de cães, após a remoção cirúrgica dos nematódeos. O estudo envolveu 15 cães com parasitismo renal por fêmeas de *D. renale*. Coletou-se amostras de urina antes da cirurgia e durante 10 dias após a nefrectomia, preconizando-se a primeira urina da manhã. Para a quantificação dos ovos, analisou-se 40 µL do sedimento urinário em triplicata. Nas análises laboratoriais, detectou-se entre 900 e > 6.000 ovos/amostra de urina em 86,7% dos cães antes da cirurgia, e em 40% dos cães no primeiro dia após o procedimento. Catorze cães (93,3%) eliminaram ovos de *D. renale* entre o primeiro e o décimo dia após a nefrectomia, sendo o maior pico de eliminação no primeiro dia. A partir desses resultados, observou-se que os cães podem eliminar ovos de *D. renale,* mesmo após a remoção dos helmintos do rim parasitado, destacando-se o ineditismo e a importância desses achados para a Saúde Única.

*Dioctophyme renale* (Nematoda: Enoplida) is a cosmopolitan nematode capable of parasitizing mammals and the majority of reported cases of dioctophymosis occurring in the canine species *Canis lupus* (Eiras et al., 2021). The helminth has zoonotic potential, as evidenced by the fact that cases of dioctophymosis have been reported in humans in various countries, including Brazil (Lisbôa, 1945; Yang et al., 2019). The severity of the parasitism is mainly related to the impairment of renal function, because the nematode compresses and destroys the parenchyma of the affected kidney (Perera et al., 2021). Parasitism can also occur in the abdominal cavity, thoracic cavity, subcutaneous tissue, and other locations (Caye et al., 2024; Eiras et al., 2021; Brunner et al., 2022).

In the South of Rio Grande do Sul, Brazil, it is important to investigate dioctophymosis in animals, given that parasitic forms of *D. renale* have been observed in the environment (Perera et al., 2017a). In that same region, third-stage (infective) larvae of *D. renale* have been detected in possible paratenic hosts (Mascarenhas et al., 2017, 2019), as have its adult forms in domestic and wild animals (Rappeti et al., 2017; Trindade et al., 2018). Therefore, the dissemination of *D. renale* eggs through the urine of parasitized animals perpetuates the life cycle of the helminth (Measures, 2001).

For individuals with renal parasitism, the recommended treatment is nephrotomy or nephrectomy of the affected kidney (Caye et al., 2024; Milech et al., 2022). However, to our knowledge, there have been no studies evaluating whether *D. renale* eggs remain in the urinary tract of parasitized animals after removal of the parasites or for how long the eggs persist there, which is likely because *D. renale* oviposition is quite intense. Knowing this information can show a general parameter of the dissemination of eggs through urine and, consequently, the level of environmental contamination caused by these eggs. Therefore, the objective of this study was to identify and quantify parasite eggs before and after nephrectomy in the urine of dogs naturally infected with *D. renale*.

A total of 15 domestic dogs with dioctophymosis were selected from among animals under routine veterinary care at the Ceval Outpatient Clinic of the *Hospital de Clínicas Veterinária da Universidade Federal de Pelotas* (HCV-UFPel, Clinical Veterinary Hospital of the Federal University of Pelotas), in the city of Pelotas, located in the Brazilian state of Rio Grande do Sul (31º 46’ 34” S, 52º 21’ 34” W). The dogs were designated patient P1 to P15. The inclusion criteria were being parasitized by *D. renale* and eliminating its eggs in urine. For egg elimination to occur, the kidney must be parasitized by at least one sexually mature female. Therefore, dogs that were parasitized only by male specimens were excluded, as were those in which *D. renale* was identified only in other anatomical regions.

Prior to surgical treatment to remove nematodes and the parasitized kidney, a urine sample was collected from each animal. After the complementary examinations had been carried out, all of the patients were referred to the HCV-UFPel for the surgical procedure. After nephrectomy, urine samples were collected daily for ten days, starting 24 h after surgery, sampling of the first morning urine being recommended. The samples were obtained through spontaneous voiding and were stored in collection bottles.

Immediately after urine collection, formalin (1:50, formalin:urine) was added as a preservative, and the samples were refrigerated until laboratory analysis. After homogenization of the samples, an average of 14 mL of each sample was processed using the centrifugal sedimentation technique (203 *g* for 5 minutes). The supernatant was then discarded, and the sediment was analyzed under light microscopy at 10× magnification. To quantify eggs, 40 µL of urinary sediment was analyzed in triplicate, the number of eggs being counted in each of the three aliquots and the sum of the three counts being determined. Exact counts of the number of eggs were made in each aliquot, up to a maximum of 2,000 eggs. When the number of eggs exceeded 2,000 eggs/aliquot, exact counting became unfeasible because of overlapping of the eggs. In those cases, the exact number of eggs was not determined and the result was expressed as > 2,000/aliquot.

Of the 15 dogs selected to be part of the study (ten females and five males), all were mixed breed. All of the dogs had a history of access to the outdoors. Because they all had been strays or had been adopted as adults, the exact age was unknown for all but one. Therefore, in accordance with the age classes described by Creevy et al. (2019), one of the animals was a puppy (6 months of age), 13 were adult dogs, and one was a senior dog. The higher occurrence in adult animals is probably due to nonspecific or absent clinical signs, leading to late diagnosis or accidental findings, as shown in the literature (Radman et al., 2017; Shakarboev et al., 2024).

The data regarding cases of dogs testing positive for dioctophymosis in the region are worrisome, mainly due to the fact that all animals diagnosed with the disease had access to the outdoors prior to diagnosis (Caye et al., 2024). That favors the dissemination of eggs in the environment through animal urine (Perera et al., 2017a; Eslahi et al., 2021), which, under the right conditions, can perpetuate the life cycle of the helminth (Mace & Anderson, 1975). Previous studies have verified that the first-stage larvae of *D. renale* reach their development in water at a temperature from 14 to 30 °C, as well as that the duration required for first-stage larval development decreases as the temperature for egg incubation increases (Mace & Anderson, 1975; Pedrassani et al., 2009). Furthermore, the great thickness of *D. renale* eggshells may contribute to their resistance to the environment. Thus, environmental contamination by *D. renale* eggs becomes an important risk factor for the infection of definitive hosts, especially humans given that *D. renale* is a nematode with zoonotic potential (Eslahi et al., 2021). Because that is to the detriment of the One Health concept, it is essential to raise awareness among the population regarding the severity of dioctophymosis in humans (Yang et al., 2019) and the high prevalence of infection with the parasite in animals (Eiras et al., 2021; Perera et al., 2021).

In all of the *D. renale*-infected animals that were included in the study, the helminths were observed in the right kidney, requiring nephrectomy of the organ, because that is the procedure indicated, especially when there is progressive destruction of the renal parenchyma (Perera et al., 2021; Shakarboev et al., 2024). In three animals, there were also parasites in ectopic locations such as the abdominal and thoracic cavities, from which they were also surgically removed by laparotomy and thoracotomy, respectively (Table 1).

**Table 1:** Quantification of female and male specimens of *Dioctophyme renale* observed in the right kidney and at other sites of infection in dogs with dioctophymosis,and total number of eggs observed in the urine of dogs parasitized by *Dioctophyme renale* in the preoperative period (POP) to the tenth postoperative day (POD 10) after the removal of helminths by nephrectomy.

Patient	Parasites in the right kidney	Parasites at other sites	Total	Preoperative period	Postoperative period									
	**(n)sex**	**(n)sex (site)**	**(n)**		**Day 1**	**Day 2**	**Day 3**	**Day 4**	**Day 5**	**Day 6**	**Day 7**	**Day 8**	**Day 9**	**Day 10**
P1	1F	0	1	4,319	768	508	232	16	0	1	0	0	0	NC
P2	1F	0	1	3,532	3	5	1	0	0	0	0	0	0	NC
P3	1F	0	1	652	381	385	251	99	25	59	1	2	0	0
P4	1F / 1M	0	2	98	6	1	0	0	0	0	0	0	0	6
P5	2F	0	2	> 6,000	> 6,000	192	3	2	6	8	11	43	3	2
P6	1F / 1M	0	2	> 6,000	> 6,000	0	0	0	0	0	0	0	0	NC
P7	1F	0	1	1,035	164	0	1	0	0	NC	0	NC	0	0
P8	1F / 2M	0	3	> 6,000	1,419	0	0	NC	0	0	1	0	NC	0
P9	2F	1M (AC)	3	> 6,000	932	24	1	0	0	0	0	0	0	0
P10	2F / 2M	0	4	> 6,000	> 6,000	0	1	1	7	83	3	0	0	0
P11	1F	0	1	> 6,000	0	0	0	0	1	0	0	3	0	5
P12	1F	0	1	1,340	NC	11	0	0	0	0	0	0	0	NC
P13	1F	0	1	> 6,000	0	0	0	0	0	0	0	0	0	0
P14	1F / 2M	1F (AC); 1F (TC)	5	3,465	> 6,000	2	NC	NC	0	0	0	0	1	0
P15	1F	4F and 2M (AC)	7	1,041	19	0	1	0	1	1	0	0	0	0
**Total**	**18F / 8M**	**8AC / 1TC**	**35**	**> 57,482**	**> 27,692**	**1,128**	**491**	**118**	**40**	**152**	**16**	**48**	**4**	**13**
**Mean**	**1.2F / 0.5M**	**0.5AC / 0.06TC**		**> 3,832**	**> 1,978**	**75.20**	**35.07**	**9.07**	**2.66**	**10.85**	**1.06**	**3.42**	**0.28**	**1.18**

F: female; M: male; AC: abdominal cavity; TC: thoracic cavity; NC: not collected.

Of the 26 helminths found in right kidneys, 18 were female (1.2 female parasites/dog) and eight were male (0.5 male parasites/dog). As can be seen in Table 1, the kidneys were parasitized exclusively by female specimens in ten (66.7%) of the dogs and by male and female specimens in the same kidney in five (33.3%). The latter condition can result in the formation of fertile eggs (Mace & Anderson, 1975). Given that the elimination of eggs through urine is necessary for the continuation of the *D. renale* life cycle (Measures, 2001), that finding makes the present study even more relevant, because the eggs present in the urinary tract continue to be eliminated by the animals even after nephrectomy. This serves as a warning that should prompt the creation of prophylactic and control measures for dioctophymosis that take the One Health recommendations into account. An integrated approach can lead to better prophylaxis against infectious agents in the environment (Ojeyinka & Omaghomi, 2024), especially those that cause zoonotic diseases such as dioctophymosis.

Infection by high numbers of *D. renale* specimens has been documented in dogs in the city of Pelotas. In one of these cases, Caye et al. (2020) identified 34 specimens in one dog in the city: seven in the right kidney and 27 in the abdominal cavity. Similarly, Perera et al. (2017b) identified 23 specimens in the abdominal cavity of a dog that had eliminated three of the helminths from its urinary tract prior to surgery. Despite the relevance of reports of high *D. renale* infection in dogs, such reports are uncommon, because *D. renale* parasitism typically involves a smaller quantity of helminths (Brunner et al., 2022). That is in line with the data obtained in the present study, in which the majority (66.7%) of the patients were infected with only one or two specimens.

Because it is a dioecious helminth, the presence of at least one pair of sexually mature *D. renale* individuals in the kidney of the definitive host is essential for the formation of fertile eggs and the continuation of the life cycle, a situation that was observed in 33.3% of the dogs in the present study. In the remaining cases, only mature females were observed in the kidney, which made it possible to identify eggs through urinalysis, although those eggs would not perpetuate the life cycle if they had not been fertilized by male helminths (Measures, 2001). However, there have been reports of parasitism by male *D. renale* specimens in the right kidney and their elimination in urine after having fertilized the females, because the males are smaller and narrower, which facilitates their passage through the urethra (Lisbôa, 1945; Measures, 2001). Therefore, the possibility of fertile eggs in cases of the absence of males during surgical removal of helminths cannot be ruled out. However, to confirm this hypothesis, it would be necessary to determine the embryonic capacity of the expelled eggs.

*Dioctophyme renale* is capable of producing thousands of eggs per day. Those eggs can also be eliminated by the thousands in the host urine, as observed in the present study. We found that 86.7% of the dogs evaluated eliminated a high number of eggs in their urine prior to nephrectomy (≥ 1,035/urine sample), including those that had only one female in the kidney (Table 1), which is highly relevant for environmental contamination. The massive dissemination of eggs is likely related to the level of sexual maturity and viability of the female specimens of the helminth, regardless of the presence or absence of a male specimen.

We confirmed that parasitized animals can continue eliminating eggs and contaminating the environment even after surgical treatment, contributing to the continuation of the *D. renale* lifecycle. Another worrisome finding is that there was the high quantity of parasite eggs (> 2,000/40 µL of urinary sediment, total of > 6,000/urine sample) in the urine of seven patients preoperatively and in that of four patients on postoperative day (POD) 1. That demonstrates the high egg-laying capacity of female specimens of *D. renale*, which leads to considerable dissemination of the eggs into the environment when the female reaches sexual maturity. In the present study, 93.3% of the dogs expelled more than 652 eggs in a urine sample on the day before surgery (Table 1). It is likely that the kidney of P2, a dog that eliminated fewer eggs (n = 98) before surgery, was parasitized by a less viable *D. renale* female.

Although the dogs evaluated eliminated a greater number of eggs immediately after surgery (on POD 1) than thereafter, urinary elimination continued until POD 5 in five dogs and until POD 10 in three. According to the daily evaluation of urine samples, the mean number of eggs eliminated was > 1,978 on POD 1, compared with only 75 on POD 2 and only 35 on POD 3 (Table 1). From POD 4 onward, the mean number of eggs expelled in the urine of the dogs was considerably lower (9 eggs/urine sample). Although this is the first study reporting the postoperative elimination of *D. renale* eggs, that finding was expected, given that the eggs detected during that period were the product of oviposition by nematodes before they were removed from the kidney. Because of the high numbers of eggs produced by *D. renale* females, some eggs remain lodged in the urinary tract (bladder and urethra) of animals after nephrectomy, subsequently being eliminated into the environment. That is relevant because each fertilized egg can become an infective larva, maintaining the parasite life cycle and triggering dioctophymosis in the definitive hosts that become infected.

In addition, we observed intermittent elimination of eggs in urine; that is, there were days with no egg detection in 53.3% of the patients, in whose urine samples eggs were again detected on subsequent days. We believe that this occurred because the *D. renale* eggs adhered to surfaces within the urinary tract, causing them to be retained and expelled intermittently in the urine. There are reports in the literature stating that the undulated surface of the shell of a *D. renale* egg can function as an attachment point for aquatic plants (Pedrassani et al., 2009). That rugosity is also thought to facilitate the adhesion of the eggs to the mucous membranes in the bladder and urethra of infected animals. It is also possible that the timing of urine collection and the amount of urine expelled influenced the intensity of egg elimination.

It should be borne in mind that the number of *D. renale* eggs eliminated is greater than that recorded in the present study, because only one aliquot of each urine sample was analyzed per patient day. Therefore, it is logical to assume that eggs were present in the rest of each sample and in the urine produced at other times of the day. That alerts us to the need to take precautions during the preoperative and postoperative periods in animals with dioctophymosis, because such animals could contaminate the environment; the fact that no eggs were seen in the urine collected at a given time point does not necessarily mean that elimination did not occur at other times. Therefore, there is a need for new studies to assess how long eggs are still eliminated in the urine so as not to interfere with urinalysis diagnostic tests.

**Figure 1:**
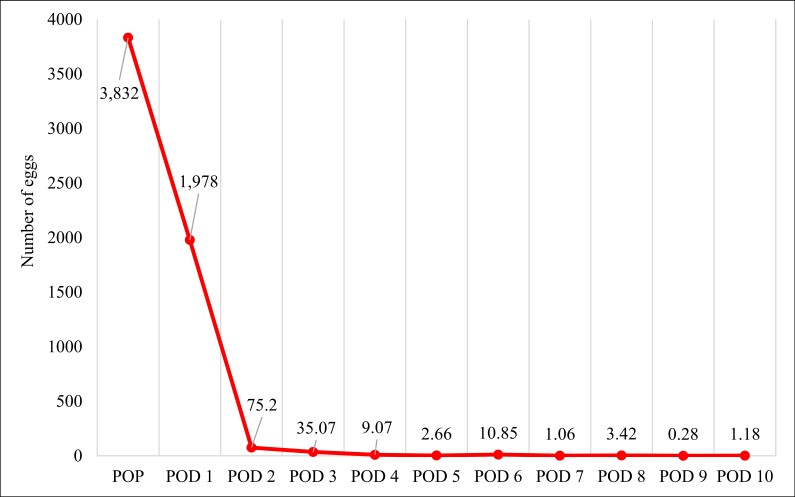
Mean number of *Dioctophyme renale* eggs eliminated in dog urine in the preoperative period (POP) to the tenth postoperative day (POD 10) after the removal of helminths by nephrectomy.

Of the patients monitored, 93.3% eliminated *D. renale* eggs in urine after the surgical procedure, with around 99% of the eggs being eliminated by POD 3 (Figure 1). To our knowledge, that is an unprecedented finding. In fact, the quantification of *D. renale* eggs eliminated in dog urine is also unprecedented, previous studies having mentioned only their presence in urine samples (Perera et al., 2017a), without specifying the number of eggs.

The elimination of *D. renale* egg speaked on the first day after surgical removal of the parasites from the kidney. The quantity of eggs eliminated in urine was considerably lower on POD 2 and POD 3 than on POD 1 (Figure 1). However, as previously mentioned, eggs were counted only in three aliquots of a 14 mL urine sample per day. Given that the physiological urinary production of dogs is 1–2 mL/kg per hour (Borin-Crivellenti & Maia, 2021), the quantities of eggs found on POD 2 and POD 3 are also relevant, because the proportion of eggs disseminated in the environment would be even greater than that. Therefore, it is recommended that such animals be contained during the postoperative period in order to reduce the spread of eggs into the environment. The urine produced by the animals during that period must be disposed of appropriately in order to prevent larval development. It has been suggested that, prior to its disposal, *D. renale*-infected urine be frozen, dried, or kept at a temperature above 33°C for at least five days (Mace & Anderson, 1975). The ideal would be to sterilize the urine by heat (autoclaving), following the standard procedure recommended for controlling microorganisms: 121°C for 20 min at 100 kPa (Aljamali et al., 2020). Because elimination of the eggs retained is greatest in the first few days after surgery, another option for control would be to flush the bladder with saline solution during surgery, after removing the parasites, in order to reduce the dissemination of eggs into the environment through the urine of the affected animals.

Our results indicate that *D. renale* eggs are eliminated in dog urine for at least ten days after the surgical removal of helminths from the kidney, the first three days after surgery being crucial. Therefore, animals with dioctophymosis can be a source of helminth infection even after surgical treatment. This information is important, not only for understanding the parasite life cycle but also for establishing means of control and prophylaxis.
